# The Role of Downsizing of Large-Bore Percutaneous Femoral Access for Pelvic and Lower Limb Perfusion in Transfemoral Branched Endovascular Aortic Repair

**DOI:** 10.3390/jcm13185375

**Published:** 2024-09-11

**Authors:** Daour Yousef Al Sarhan, Tilo Kölbel, Alessandro Grandi, Petroula Nana, José I. Torrealba, Christian-Alexander Behrendt, Giuseppe Panuccio

**Affiliations:** German Aortic Centre, Department of Vascular Medicine, University Medical Center Hamburg-Eppendorf, 20251 Hamburg, Germany; d.yousef-al-sarhan@uke.de (D.Y.A.S.); tilokoelbel@googlemail.com (T.K.); alex@eenet.it (A.G.); jitorrelba@gmail.com (J.I.T.); behrendt@hamburg.de (C.-A.B.); g.panuccio@uke.de (G.P.)

**Keywords:** transfemoral access, branched endovascular aortic repair, sheaths, downsizing, lower limb perfusion, complications

## Abstract

**Background**: Transfemoral access (TFA) is a valuable alternative to upper extremity access (UEA) for branched endovascular aortic repair (bEVAR). However, TFA requires large introducer sheaths, which can reduce blood flow to lower limbs and the pelvis. This study aimed to evaluate the efficacy of sheath downsizing to maintain lower limb perfusion during TFA–bEVAR. **Methods**: A single-center retrospective review was conducted including patients managed with TFA-performed bEVAR between December 2020 and May 2021. Intra-operative lower limb perfusion was assessed using non-invasive ankle blood pressure measurements and great toe pulse oximetry, with measurements being taken prior to puncture (baseline), one minute after 10F-sheath insertion, three minutes after the main body delivery system insertion, and three minutes after downsizing to a 14F sheath. Outcomes included the incidence of limb perfusion reduction (LPR), defined as a drop in the ankle–brachial index (ABI) < 0.5 or peripheral oxygen saturation (SpO_2_) < 90%. **Results**: Out of 47 patients, 24 met the inclusion criteria. LPR occurred in 4.2% of cases after 10F-sheath placement, and 87.5% after main body delivery system placement, and decreased to 12.6% after downsizing to a 14F sheath. No periprocedural major bleeding occurred. Two patients required revision for inadequate hemostasis post-operatively. SCI occurred in 16% of patients, all recovered by discharge. Pre-operative hypogastric artery occlusion was related to persistent LPR after downsizing (100% vs. 16%, *p* = 0.009). **Conclusions**: Downsizing the introducer sheath during bEVAR is feasible and safe to restore lower limb and pelvic perfusion. Further research is needed to clarify the access downsizing value during bEVAR.

## 1. Introduction

In recent years, transfemoral access (TFA) has emerged as a viable alternative to upper extremity access (UEA) for the catheterization of antegrade branches during branched endovascular aortic repair (bEVAR) [1,2]. The primary impetus for this shift to TFA is the significant risk of complications associated with UEA, including stroke, local vessel and nerve injury, pseudoaneurysm formation and bleeding at the access site [3,4,5,6]. Especially for the right or bilateral UEA, the complication rate is estimated as five times higher compared to left UEA, including a higher risk for stroke [6]. Conversely, previous studies have demonstrated that TFA is associated with reduced radiation exposure and fluoroscopy time, decreased blood loss, shorter operative time, and lower stroke rates, especially regarding the steps related to target vessel catheterization and stenting during bEVAR [7,8,9].

However, the main drawback of TFA is the need to keep a transfemoral large-bore access sheath in place, not only during the main endograft deployment but also until all target vessels have been catheterized and bridged. This may restrict the distal blood flow in both lower limbs and the hypogastric circulation and further lead to spinal cord ischemia (SCI) evolution [4,10,11]. To overcome this pitfall of TFA, a downsizing of the sheath used for main vascular access has been previously described, with high technical success and safety rates [12]. This technique allows the operator to exchange the large-bore sheath required for bEVAR (18–23F) for a non-occlusive introducer sheath (14F) and restore the flow to the lower limbs and pelvis. After deployment of the main aortic endografts, the introduction system is exchanged for a 14F sheath and the sutures of the vessel closure device (VCD), which has been previously implanted, are tightened until no bleeding is observed [12]. Additional VCD should be available in the operating theater, as previous data showed that the use of additional devices may be needed at the completion of the procedure to achieve complete access site hemostasis [12]. Despite the rational hypothesis that a decreased-diameter sheath may increase the blood flow to lower limbs and hypogastric circulation, data on the clinical impact and distal perfusion after sheath downsizing are completely lacking.

Thus, the aim of the present study was to explore the efficacy of the downsizing technique in preserving lower limb perfusion during bEVAR with TFA, by estimating the changes in the ankle–brachial pressure index (ABPI) and peripheral oxygen saturation (SpO_2_) of the lower limb during the different phases of the procedure.

## 2. Materials and Methods

### 2.1. Study Cohort

**Figure 1:**
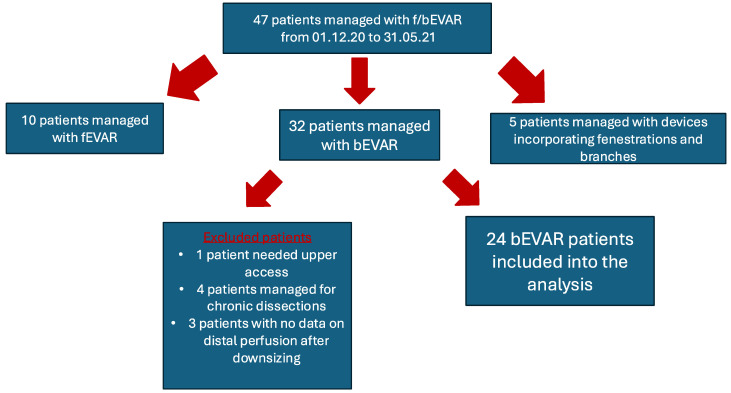
Among 47 patients managed from December 2020 to May 2021 with fenestrated or branched endovascular aortic repair, 32 patients were managed with branched devices. However, 24 fulfilled the predefined criteria and were included in the current analysis.

This study was conducted following the STrengthening the Reporting of OBservational studies in Epidemiology (STROBE) guidelines [13]. Patients undergoing complex endovascular aortic repair with bEVAR using exclusively TFA and sheath downsizing intra-operatively, from 1 December 2020 to 31 May 2021, at a single large-volume aortic center were considered eligible and retrospectively reviewed. The extent of the disease was not a criterion for exclusion and patients with degenerative pararenal and thoracoabdominal (TAAA) aneurysms type I-IV, according to modified Crawford’s criteria, were included (Figure 1) [14]. Any previous endovascular intervention, including proximal thoracic endovascular aortic repair (TEVAR), to provide adequate proximal aortic landing, was not a criterion for exclusion while downsizing during standard TEVAR was not performed, due to the nature of the procedure. Intra-operative lower limb perfusion assessment had been routinely performed during this period and data on both ABPI and SpO_2_ of the lower limbs were collected and recorded. Only patients with available data were considered eligible (three patients were excluded due to incomplete records of ABPI and/or SpO_2_). Patients managed with fEVAR or devices simultaneously incorporating fenestrations and branches were excluded due to differences in the applied technique and in order to increase the homogeneity of the cohort. This study complied with the Declaration of Helsinki, and ethical approval from the local ethics committee was not deemed necessary due to its retrospective design and unidentifiable information, according to the current state law.

Both off-the-shelf and custom-made devices relying exclusively on the Zenith platform (Cook Medical, Bjaeverskov, Denmark) were used in all cases. The procedures were performed in a dedicated hybrid operating room with a fixed imaging system and computed tomography angiography (CTA) fusion imaging (Vessel Navigator; Philips Healthcare, Best, The Netherlands). Systemic heparinization was used in all patients, with an activated clotting time goal at 250–350 s. The operative technique has been previously described [7]. To reduce the risk of SCI, a specific intra- and post-operative preventive protocol with mean arterial blood pressure ≥ 90 mmHg, central venous saturation ≥ 70%, central venous pressure ≤ 10 mmHg, and the peri-operative hemoglobin threshold at 8 mg/dL was applied [15]. The use of prophylactic cerebrospinal fluid drainage (CSFD) depended on the anatomy and history of the patient [15]. Patients with a compromised collateral network (stenosis or the occlusion of the left subclavian and/or hypogastric arteries), as well as patients needing extensive aortic coverage of more than 200 mm and/or presenting with an anamnesis of previous aortic repair, open or endovascular, were considered candidates for prophylactic CSFD [15]. Therapeutic CSFD was used in all symptomatic SCI cases.

### 2.2. Access Technique and Downsizing

All procedures were performed using ultrasound-guided percutaneous access from the right or left common femoral artery (CFA) and iliac axis, using a single VCD (Prostar XL, Abbott, IL, USA) and the initial introduction of a short (11 cm) 10F sheath [16,17]. The decision to use the right or left CFA depended on the anatomy of the patient. Right CFA was preferably used to introduce the aortic endograft components. In case of severe stenosis or the calcification of the iliac axis, the largest iliac vessel was chosen as the main route. In case of a unilateral occlusion of the hypogastric artery, this side was selected for main access and aortic device deployment, to permit the continuous perfusion of the spinal cord collateral network through the contralateral patent hypogastric artery. The aortic endograft and ipsilateral iliac limb were consecutively introduced and deployed, followed by the contralateral limb. Subsequently, the large-bore introduction system was exchanged for a 14F × 45 cm sheath, which would house a modified, using a 0.014’ wire, steerable sheath for antegrade branches and target vessels’ catheterization, as well as bridging stent advancement and deployment [7]. To avoid back-bleeding from the percutaneous TFA after downsizing the introduced sheath, traction was applied on the sutures of the previously placed VCD, until adequate hemostasis was achieved. After the completion of the procedures, the sheaths were retrieved, and the access site was closed. Manual pressure was applied for 5–10 min.

### 2.3. Parameters’ Assessment

To monitor the lower limb perfusion of the main access side during the procedure, a non-invasive blood pressure measurement at the ankle and pulse oximetry on the great toe were recorded using a monitor (Infinity^®^ Delta Monitor, Draeger, Lübeck, Germany). Measurements taken prior to skin puncture were considered as baseline values. During the procedure, three distinct phases were identified, and measurements were analyzed at the following phases: one minute after the insertion of a 10F introducer sheath following VCD placement (phase 1); three minutes after the insertion of the main body delivery system (phase 2); and three minutes after downsizing and placement of the 14F sheath (phase 3). Measurements of the contralateral access site were not performed, as no large-bore sheath (≥18F) was used, and no downsizing was performed. At the same time, invasive blood pressure from the radial artery and pulse oximetry of the upper limb were recorded for ABPI calculation and as control values.

### 2.4. Data Collection

Pre-, intra-, and post-operative information was collected and introduced in a common database, including demographics, comorbidities, anatomic information, and values of ABPI and SpO_2_ at the three distinct intra-operative phases. The duration of each phase was also recorded. Measurements at the end of the procedure were also collected. The rate of access bleeding during the downsizing phase as well as the need for surgical revision at the end of the procedure were recorded. Common and external iliac arteries’ diameters were measured, and the presence of hypogastric artery occlusion was recorded, according to the pre-operative computed tomography angiographies (CTAs), in order to evaluate any potential correlation of their diameter or patency state to limb perfusion reduction (LPR) recovery during the procedure. The incidence of SCI during the peri-operative period and until 30 days after the procedure was recorded. SCI complete, partial, or no recovery were noted.

### 2.5. Definitions

Limb perfusion reduction (LPR) was defined as a drop in the ABPI below 0.5 or a drop in the peripheral SpO_2_ of the great toe below 90% [10,18]. If SpO_2_ or blood pressure measurement was not possible because it was below the sensitivity range of the sensor, this was also considered as LPR. SCI was considered as any new-onset post-operative neurologic lower limb deficit, not attributable to any other pathology, up to 30 days after the procedure [19]. No further SCI analysis was attempted in terms of time of evolution or grade due to the limited number of cases. Major bleeding was considered any obvious sign of bleeding requiring at least medical intervention, leading to longer hospitalization or an increase in the level of care [20].

### 2.6. Outcomes

The main outcome of this study was the incidence of limb perfusion reduction (LPR) during the three intraprocedural phases. In addition, a comparative analysis was performed among patients that presented LPR during phase 2 and showed persistent vs. recovered LPR in phase 3, in order to investigate the role of common and external iliac artery diameter, hypogastric artery occlusion, and SCI with the incidence of LPR after downsizing.

### 2.7. Statistical Analysis

Continuous variables were expressed as the median with IQR, due to the limited sample size. Differences between continuous variables were tested with the Mann–Whitney U test. Categorical variables were expressed as counts and percentages and the Chi-square or Fischer’s exact test was used for comparisons. No regression analyses were attempted due to the small cohort size and the lack of adverse events. A *p* value was considered significant when <0.05. The sample size was allowed to vary based on the analysis, and no imputation of missing data was performed, as an absence of variables was infrequent (<5%). Statistical Package for the Social Sciences (SPSS, Version 12.0, SPSS, Inc., Chicago, IL, USA) software for Windows was used for the data analysis.

## 3. Results

### 3.1. Patient Cohort

During the study period, 47 patients were managed for f/bEVAR; 24 had available ABPI and SpO_2_ measurements and were included in the present study. Seventeen patients were males (71%) and the median age of the cohort was 69 years (64–78 years). The most common comorbidity was hypertension, affecting 75% of the cohort while four patients (17%) had a known history of peripheral arterial disease (PAD). Regarding the aortic lesions treated among the cohort, three patients (13%) were managed for type I TAAA, six (25%) for type II, three (13%) for type III, and two (8%) for type IV. The remaining ten patients (42%) were managed for a pararenal aortic aneurysm. Details on the pre-operative baseline patient characteristics are reported in Table 1.

The median sheath size of the aortic main bodies was 22F (range: 20–22F). The median diameter of the common iliac artery was 1.3 cm (range: 1.2–1.4 cm) and for the external iliac artery, it was 1.0 cm (0.9–1.1 cm). In all cases, the sheath size was smaller than the common (median sheath to artery ratio: 0.49, 0.44–0.52) and external iliac artery (median sheath to artery ratio: 0.73, 0.66–0.81).

### 3.2. Limb Perfusion Reduction

In Table 2, the ABPI and SpO_2_ are presented, in addition to the LPR incidence and duration of the intra-operative phases. No patient presented signs of compromised limb perfusion at the beginning of the procedure (median ABPI: 1.05 and median SpO_2_: 100%). Only one patient presented with LPR (4.2%) after the placement of the 10F sheath in the groin (phase 1) compared to 21 cases (87.5%) presenting with LPR after the introduction of the aortic main body sheath (phase 2). During phase 3, after downsizing to a 14F sheath, only three cases presented with persistent LPR (12.6%). At the end of the procedure, all LPR events were restored and ABPI and SpO_2_ were higher than 0.5 and 90%, respectively. Phase 3, which included TV catheterization and bridging stent advancement and deployment, was the most time-consuming with a duration of 125 min compared to 20 min for phase 1 and 30 min for phase 2.

There was no peri-operative major bleeding related to vascular access during the downsizing phase. After CFA closure with the VCD, two patients required a surgical revision of the groin due to insufficient hemostasis at the completion of the procedure. Both cases were managed adequately without further complications or reinterventions regarding access at 30 days. SCI occurred in four cases (16%) during the early post-operative period. All SCI patients had fully recovered at discharge.

Among 21 patients that presented LPR during phase 2, 18 patients with a persistent LPR vs. 3 with recovered LPR during the downsizing phase (phase 3) were compared in terms of EIA and CIA diameters, hypogastric artery occlusion, and SCI evolution. EIA and CIA diameter comparison showed no statistically significant difference. Within the same sub-cohort, pre-operative hypogastric artery occlusion was more frequent in patients with a persistent LPR (100% vs. 16%, *p* = 0.009). The incidence of SCI was higher among patients with persistent LPR compared to patients with recovered LPR (14.3% vs. 33%), but did not achieve statistical significance, as presented in Table 3.

## 4. Discussion

The use of exclusive TFA during complex endovascular procedures, including the thoracoabdominal aorta and aortic arch, has been previously reported, and has been related to higher technical success, lower radiation exposure for the operator, and decreased operative time, in addition to lower access complication and stroke rates compared to the use of an additional upper limb access [7,21,22,23]. However, no data were available studying the correlation between lower limb and pelvic reperfusion after sheath downsizing, especially in terms of clinical measurements [7,21]. The present study demonstrates a recovery from LPR following downsizing of the introducer sheath from 87.5% during aortic graft deployment to 12.6% during branch and target vessel catheterization, which represent the most time-consuming phase of the procedure. Although the incidence of LPR is probably higher than the incidence of pelvic perfusion reduction, it can be hypothesized that lower limb perfusion is an indirect marker of pelvic flow improvement during complex endovascular aortic procedures. In clinical aspects, the rate of SCI was lower among patients with LPR recovery, though without achieving significance potentially due to the small sample size and limited number of adverse events. The presence of an occluded hypogastric artery was related to persistent LPR and may have been related to a compromised collateralization of the pelvic and deep femoral artery.

The role of VCDs seems mandatory to perform access downsizing during bEVAR and prevent bleeding [21]. VCDs can be grouped into two different types: suture-based and plug-based. Crucial for a downsize technique is the ability to reduce the access size similar to the well-known technique of purse-string sutures performed during CFA cut-down. Melloni et al. introduced and described the potential role of downsizing a percutaneous access, using a suture-based VCD, to reduce limb ischemia time [12]. However, lower limb perfusion was not measured in that study [12]. Previous studies also suggested the use of downsizing the introducer sheath, which would permit sheath retrieval below the ipsilateral hypogastric artery but reported the sheath adjustment in combination with CFA cut down or the use of conduits [10,24].

The VCD used in the current analysis has two braided non-resorbable sutures and has been related to high effectiveness and safety for percutaneous closure of femoral access when large-bore sheaths are used [25]. During suture placement, four needles simultaneously cross the vessel upper wall from the inside to the outside, reducing the risk of dissection. The precise orientation and non-elastic type of this braided suture allows potentially better control of the suture tension, enabling successful downsizing. Similarly, recent data showed that effective hemostasis and lower access complication rates can be achieved during up- or downsizing of a large introducer sheath using a single suture-based technique [26]. Suture-based VCDs seem to provide similarly good outcomes when performing sheath downsizing according to the limited literature [12,26]. As our main experience is represented using the Prostar XL (Abbott, IL, USA) VDC, comparative information could not be provided in this analysis, and it suggests that a VDC over another would be based only on hypotheses compared to real data. The familiarity of the operator with a VCD and knowledge of its potential benefits and pitfalls are crucial for decision making during VCD selection to perform sheath up- and downsizing.

It should be noted that in the current analysis, while intra-operative hemostasis during downsizing was possible in all cases, two patients needed an open conversion of the access site to achieve complete hemostasis after the completion of the procedure. Small femoral arteries and severe access vessel calcifications have been previously detected as predictors of failure during the Perclose technique for sheaths larger than 21F [27]. Additional VCDs should be available at any time in the operating theater as early post-operative bleeding cannot be excluded, even when appropriate hemostasis has been achieved intra-operatively [12].

Early limb reperfusion is important as catheterization times in f/bEVAR can be time- and radiation-consuming, even with TFA [4,7]. Phase 3, which includes TV catheterization and bridging, and is performed under sheath downsizing, was the most time-consuming compared to the other two phases in this analysis. Considering the profile of the main aortic graft components being 4 to 8F bigger than the sheath used to access and catheterize the TVs, downsizing may restore limb and hypogastric perfusion in most cases and significantly decrease the risk of SCI evolution, from 25% to 2.1%, even in extensive TAAAs type I–III [11,28]. Recent data applying sheath downsizing as a standard approach during complex endovascular procedures showed a significant reduction in SCI rates [29]. Despite that no dedicated sheaths are currently available for transfemoral TV catheterization and bridging-covered stent advancement and deployment, surgeon-modified and commercially available steerable sheaths seem to provide a reliable solution and decrease the operative time during f/bEVAR procedures [30,31,32,33]. Future dedicated sheaths may further speed up catheterization times and reduce access size during this phase of f/bEVAR [31].

A significative correlation between hypogastric artery occlusion and LPR was found despite the small sample size and may represent an indirect indicator of peripheral arterial disease (PAD) of the lower limb. However, main access from the side with hypogastric occlusion was intentionally used, to permit continuous spinal cord perfusion through the collateral network of the contralateral side during the procedure. Limb reperfusion in these cases is mandatory in order to decrease tissue ischemia, oxidative stress, and reperfusion effects, which has been related to homeostasis instability and renal injury in surgical patients [34,35]. While UEA may represent a valid alternative to TFA in patients with PAD, in order to avoid prolonged lower limb ischemia, the higher stroke risk accompanying UEA should also be taken into consideration in these cases, especially when atherosclerosis affects the aortic arch and supra-aortic trunks [36,37,38]. The careful evaluation of the lower extremity access vessels before the procedure using adequate imaging remains essential in the procedural planning of such individuals, while intra-operative puncture under ultrasonographic guidance is mandatory to avoid occlusive or hemorrhagic complications and early reinterventions.

Future studies focusing on the hemodynamic alterations and clinical impact of early limb reperfusion are needed to evaluate the role of sheath downsizing during complex endovascular procedures and its potential benefits in SCI prevention. The use of invasive blood pressure measurements of the lower limb, calculation of the toe–brachial index (instead of ABPI), and use of near-infrared spectrometry could provide reliable information on the pelvic ischemia and restoration of flow after sheath downsizing during bEVAR. However, the invasiveness and associated costs of these methods set the need for specific prospective protocols and better financial resources. Expanding the population of the current cohort and applying a similar protocol of the non-invasive estimation of the lower limb blood flow could also assist the verification of our findings. For the moment, introducer sheath downsizing is used as a measure of good clinical practice in large aortic centers during complex endovascular aortic procedures and may have played a role in the decreasing rates of SCI through time [29].

### Limitations

Several limitations should be considered while interpretating the findings of the current study. First, the small sample size may not be representative of a real-world cohort and may introduce selection bias. Among 47 patients managed with fenestrated or branched endovascular repair during the study period, 24 were included in the current analysis, representing 51.1% of all patients and 75% of patients managed with bEVAR. This fact could reinforce any potential selection bias. ABPI and SpO_2_ measurements performed in the present study do not directly reflect the hypogastric perfusion but represent a non-invasive indirect marker of the lower limb and pelvic perfusion. Furthermore, non-invasive blood pressure measurement at the ankle is not very sensitive to absolute ischemia. The use of near-infrared spectrometry applied in the gluteal region may have given better information on pelvic ischemia. Unfortunately, this technology is expensive and potentially associated with imaging artifacts during the procedure. Furthermore, this study was performed at a referral center for complex endovascular procedures with high expertise with the use of VCD, and therefore the results may not be reproducible. Previous data showed an association between SCI and early hypogastric reperfusion. Despite that the SCI rate in the current study was lower among patients with restored LPR, statistical significance was not achieved. Type II statistical errors should be taken into consideration when evaluating the outcomes of the current analysis. However, the limited number of adverse events and the small cohort size did not permit further reliable uni- or multivariate analyses.

## 5. Conclusions

The downsizing technique during transfemoral bEVAR appears to be promising, safe, and feasible for maintaining and restoring the perfusion of the lower limbs and pelvic circulation and potentially decreases the spinal cord ischemia rate during complex endovascular procedures. Further studies of larger populations and potentially different modalities for pelvic perfusion calculation are needed to confirm the findings of this analysis.

## Figures and Tables

**Table 1:** Pre-operative patients’ comorbidities and baseline anatomic characteristics, regarding aneurysms’ extent.

Variable	Total n = 24
Age, years	69 (64–78)
Male, n (%)	17 (71)
Coronary artery disease, n (%)	9 (38)
Atrial fibrillation, n (%)	5 (21)
Myocardial infarction, n (%)	3 (13)
Chronic heart failure, n (%)	5 (21)
Hypertension, n (%)	18 (75)
Dyslipidemia, n (%)	4 (17)
Smoker, n (%)	7 (29)
Chronic obstructive pulmonary disease, n (%)	2 (8)
Diabetes, n (%)	2 (8)
Renal insufficiency, n (%)	9 (38)
Basal glomerular filtration rate, mL/min	53 (29–74)
Stroke, n (%)	2 (8)
Peripheral artery disease, n (%)	4 (17)
Body mass index, kg/m^2^	25 (23–29)
Aneurysm type	
Type I, n (%)	3 (13)
Type II, n (%)	6 (25)
Type III, n (%)	3 (13)
Type IV, n (%)	2 (8)
Pararenal, n (%)	10 (42)

**Table 2:** Summary of intra-operative limb perfusion reduction-related outcomes by phase. TV: target vessel; ABPI: ankle–brachial pressure index; F: French; IQR: interquartile range; LPR: limb perfusion reduction; n: number; NR: not recorded. SpO_2_: peripheral oxygen saturation.

	Baseline	Phase 1	Phase 2	Phase 3	Postop
Phase description		Access preparation	Branched graft deployment	Transfemoral TV catheterization and stenting	
Sheath size (F)		10F	20F–22F	14F	
Duration in min, Median (IQR)	NR	20 (15–30)	30 (15–30)	125 (105–190)	NR
ABPI, Median (IQR) [n]	1.05 (1–1.13) [24]	0.96 (0.86–1.08) [23]	0.63 (0.6–0.88) [3]	0.78 (0.63–0.83) [21]	1.01 (0.95–1.09) [24]
SpO_2_ (%), Median (IQR) [n]	100 (100–100) [24]	100 (97–100) [23]	97 (71–99.5) [4]	100 (99–100) [21]	100 (100–100) [24]
LPR incidence, [n] %		1 4.2	21 87.5	3 12.6	0 0.0

**Table 3:** Comparative analysis among patients with recovered LPR (n= 18) vs. patients with persistent LPR (n = 3) during phase 3. Pre-operative anatomic characteristics and post-operative SCI evolution were compared between these two groups. Footnotes—CIA: common iliac artery; EIA: external iliac artery; HA: hypogastric artery; LPR: limb perfusion reduction; n: number; SCI: spinal cord ischemia.

	Recovered LPR	Persistent LPR	*p*
n (%)	18 (85.7%)	3 (14.3%)	
EIA diameter (cm)	0.95 (IQR: 0.9–1.1)	0.9 (IQR: 0.8–1.0)	0.32
CIA diameter (cm)	1.3 (IQR: 1.2–1.4)	1.3 (IQR: 1.2–1.5)	0.81
HA occlusion	3 (16.7%)	3 (100%)	0.009
SCI	3 (16.7%)	1 (33%)	0.49

## Data Availability

Data are available upon reasonable request from the corresponding author.
